# Hypercalcemia Associated with Calcium Supplement Use: Prevalence and Characteristics in Hospitalized Patients

**DOI:** 10.3390/jcm4030414

**Published:** 2015-03-09

**Authors:** Maria C. Machado, Araba Bruce-Mensah, Melanie Whitmire, Ali A. Rizvi

**Affiliations:** 1 Division of Endocrinology, University of South Carolina School of Medicine, Columbia, SC 29203, USA; E-Mail: Maria.Machado@uscmed.sc.edu; 2 University of South Carolina School of Medicine, Columbia, SC 29203, USA; E-Mail: Araba.Bruce-Mensah@uscmed.sc.edu; 3 Research Unit, Department of Internal Medicine, University of South Carolina School of Medicine, Columbia, SC 29203, USA; E-Mail: Melanie.Whitmire@uscmed.sc.edu; 4 Department of Medicine, University of South Carolina School of Medicine, Columbia, SC 29203, USA

**Keywords:** hypercalcemia, renal insufficiency, calcium, milk-alkali syndrome

## Abstract

Background: The ingestion of large amounts of milk and antacids to treat peptic ulcer disease was a common cause of hypercalcemia in the past (the “milk-alkali syndrome”). The current popularity of calcium and supplements has given rise to a similar problem. Objectives: To evaluate the prevalence and characteristics of hypercalcemia induced by calcium intake (“calcium supplement syndrome”; or CSS) in hospitalized patients. Methods: We conducted a retrospective; electronic health record (EHR)-based review of patients with hypercalcemia over a 3-year period. Diagnosis of CSS was based on the presence of hypercalcemia; a normal parathyroid hormone (PTH) level; renal insufficiency; metabolic alkalosis; a history of calcium intake; and documented improvement with treatment. Results: Of the 72 patients with non-PTH mediated hypercalcemia; 15 (20.8%) satisfied all the criteria for the diagnosis of CSS. Calcium; vitamin D; and multivitamin ingestion were significantly associated with the diagnosis (*p* values < 0.0001; 0.014; and 0.045 respectively); while the presence of hypertension; diabetes; and renal insufficiency showed a trend towards statistical significance. All patients received intravenous fluids; and six (40%) received calcium-lowering drugs. The calcium level at discharge was normal 12 (80%) of patients. The mean serum creatinine and bicarbonate levels decreased from 2.4 and 35 mg/dL on admission respectively; to 1.6 mg/dL and 25.6 mg/dL at discharge respectively. Conclusion: The widespread use of calcium and vitamin D supplementation can manifest as hypercalcemia and worsening of kidney function in susceptible individuals. Awareness among health care professionals can lead to proper patient education regarding these health risks.

## 1. Introduction

The milk-alkali syndrome (MAS) is a disorder induced by intake of large amounts of milk, calcium preparations, and alkali for gastric acid neutralization and treatment of ulcer disease [1]. The hallmarks of MAS consist of hypercalcemia, metabolic alkalosis with elevated serum bicarbonate, and renal insufficiency [2]. Although it was a common cause of hypercalcemia in the past, the incidence of MAS reduced considerably with the decrease in popularity of traditional therapies for ulcer disease. The disorder was a cause of less than one percent of cases of hypercalcemia by the mid-1980s [3]. However, the past decade has seen a progressive reemergence of a slightly different version of MAS with its own epidemiologic and clinical characteristics [4,5,6]. The current day version is a manifestation of hypercalcemia and attendant laboratory abnormalities in predisposed individuals stemming from the widespread use of calcium and vitamin D therapy for osteoporosis or health maintenance [7,8], and therefore is more aptly labeled the “calcium supplement syndrome” (CSS). It is thought to be a relatively important cause of current-day hypercalcemia (12%), exceeded in frequency only by primary hyperparathyroidism and malignancy [7,9]. The factors leading to this change in presentation include the growing popularity of calcium intake for the maintenance of bone and musculoskeletal health and the prevention and treatment of osteoporosis.

## 2. Aims and Methods

This was a retrospective study utilizing the review of medical records of patients hospitalized over a three-year period. The study was approved by the Institutional Review Board, Palmetto Health Richland, Columbia, South Carolina, USA. The primary objective was to calculate the proportion of patients with non-parathyroid hormone (PTH)-dependent hypercalcemia that satisfied the criteria for CSS. The secondary objectives were to: (1) define the characteristics and components of CSS; and (2) describe the change in serum calcium and response to treatment during hospital stay.

**Figure 1:**
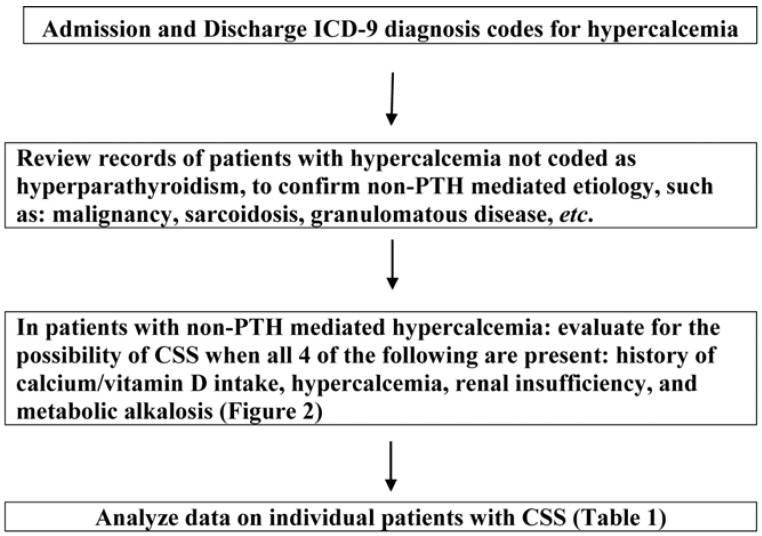
Study flow design.

**Figure 2:**
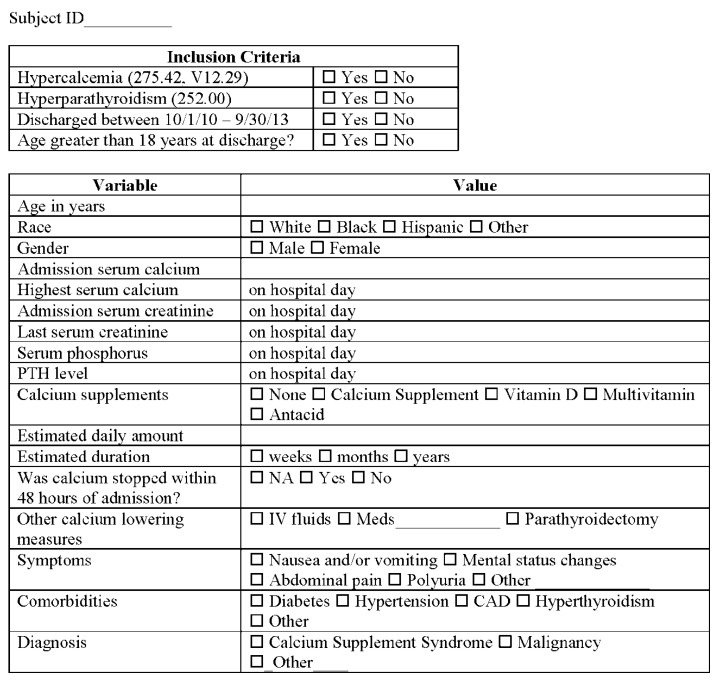
Data abstraction for individual subjects with hypercalcemia.

A flow design of the proposed study is presented in Figure 1. Data were collected in a confidential manner and duly de-identified, without accessibility to patient identifiers. Information on admission and discharge ICD-9 diagnosis codes for hypercalcemia (275.42, V12.29) was obtained on all patients above age 18 years admitted to Palmetto Health Richland over a 3-year period (1 October 2010 to 30 September 2013). The records of patients with hypercalcemia (275.42, V12.29) were reviewed to ascertain if parathyroid hormone (PTH) levels were available. Those with elevated PTH level were assumed to have PTH-mediated hypercalcemia (the majority of which had primary hyperparathyroidism or PHP). In the remaining subjects, those with a specific non-PTH mediated etiology, such as malignancy, sarcoidosis, granulomatous disease, *etc.* were excluded. In the remaining subjects, a presumptive biochemical and clinical diagnosis of CSS was made on an individual basis (Data Abstraction Tool, Figure 2). The data was entered into a Microsoft Excel spreadsheet for analysis.

## 3. Data Analysis

To calculate and justify sample size, the output from PASS 2008 gave 95% confidence interval widths for sample sizes in the anticipated range of 100 to 200 and proportions in the anticipated range of 0.05 to 0.20. The proportion of patients with hypercalcemia that satisfied the criteria for CSS was computed by dividing the number of patients that meet the criteria for CSS by the number of patients that meet the inclusion criteria. Exact (Clopper-Pearson) 95% confidence limits on this proportion were computed using the R function exactci in the PropCIs package. Serum calcium, bicarbonate and creatinine levels at admission and discharged were collected. A descriptive analysis was undertaken to define the characteristics and components of CSS and response to treatment during hospital stay. The *p* value for statistical significance for the presence or absence of CSS and the following variables was calculated: gender, calcium intake, vitamin D intake, multivitamin use, hypertension, diabetes, and renal insufficiency (as defined by a serum creatinine above 1.3 mg/dL).

## 4. Results

A total of 429 patients with hypercalcemia were identified over the three year period. Sixty-one patients had elevated calcium levels and/or a known history of PHP, while in 296 patients no PTH level was available; they either had a known non-PTH mediated cause, or the hypercalcemia was transient and was not evaluated further. The medical records of the remaining 72 patients who had either a normal or low PTH level were reviewed to further exclude specific causes of hypercalcemia, and to evaluate whether the presentation and the clinical and biochemical features of individual patients could be attributable to CSS. In individual patients, a diagnosis of CSS was ascertained based on the presence of hypercalcemia (normal range 8.5–10.1 mg/dL), a normal or suppressed PTH level (normal range 10–65 pg/mL), renal insufficiency with elevated serum creatinine (normal range 0.6–1.3 mg/dL), and metabolic alkalosis with elevated serum bicarbonate (normal range 21–32 mmol/L), and supported by a history of prescription or over-the-counter intake of calcium.

Seventy-two of the 429 patients (16.8%) were identified with hypercalcemia accompanied by a normal or low PTH. Those in whom the hypercalcemia and the clinical presentation were felt to be on the basis of a known cause, could be attributed to a specific etiology, or who did not fulfill the above-mentioned criteria for CSS, were excluded from the final analysis.

Of the 72 patients with non-PTH mediated hypercalcemia, 15 (20.8%) satisfied all the criteria for the diagnosis of CSS. Their characteristics are shown in Table 1. The mean age was 70.4 years, with a range from 54 to 87 years. There were 9 females (8 white and 1 black) and 6 males (3 white and 3 black). Fourteen (93.3%) patients had a history of hypertension and 9 (60%) had diabetes. Four patients carried a previous diagnosis of renal insufficiency. Only one patient (#12 in Table 1) was taking a thiazide diuretic. The mean serum calcium on admission was 13.3 mg/dL (range, 10.7 to 16.2), and at discharge was 9.26 mg/dL (range, 7.6 to 10.4). None of the patients had a known history of hypercalcemia in the past. The calcium level at discharge was normal in the majority (12 of the 15, or 80%) of patients, the highest level being 10.4 mg/dL. The mean PTH was 9.42 pg/mL (range, <2.5 to 53.1). The mean phosphorus level was 3.32 mg/dL (range, 2.1 to 4.6). The mean serum creatinine on admission was 2.4 mg/dL (range, 0.9 to 3.9), and at discharge was 1.6 mg/dL (range, 0.7 to 3.2). The creatinine level was normal in 7 of 15 (46.7%) patients at discharge. The mean serum bicarbonate level was 35 mg/dL (range, 32 to 43) on admission and 25.6 mg/dL (range, 17 to 33) at discharge, when it was within the normal range in all but one patient. Thus, resolution of CSS was evident by normalization or improvement in the abnormal biochemical parameters during hospital stay.

Although almost all patients suffered from cardiovascular, metabolic, and other chronic health conditions, 12 of 15 (80%) presented with complaints and manifestations consistent with, or for the most part felt to be due to, hypercalcemia (for example, weakness, fatigue, polyuria, abdominal pain, nausea, vomiting, constipation, mental status changes, and musculoskeletal discomfort). With regard to therapy, all 15 patients received rehydration with intravenous fluids, while 6 of 15 (40%) were treated with specific calcium-lowering drug treatment for the elevated calcium (4 with calcitonin and 2 with zoledronic acid).

All 15 patients were taking calcium supplements, while 12 (80%) were taking vitamin D supplements, 8 (53.3%) multivitamin tablets, and 2 (13.3%) calcium-containing antacids. Our retrospective review was unable to determine exact doses of these supplements in most patients, or whether they were being taken as over-the-counter or by prescription. It was also difficult to ascertain if the supplementation was for “health maintenance” (either self-medicated or on the advice of a health care provider) or as therapy for a specific medical condition such as osteoporosis or vitamin D deficiency. Calcium was not listed on any of the patients’ list of discharge medications. The possibility of calcium supplement-related hypercalcemia had been entertained in 5 patients by the treating physicians, although we could not find documentation of specific advice to patients to avoid excessive calcium and/or vitamin D intake.

Table 2 lists and their association with the diagnosis of CSS in our study. A history of calcium intake, presumably for health maintenance or treatment of bone disease, displayed the strongest association (*p* < 0.0001), while vitamin D and multivitamin ingestion were also significantly associated (*p* values 0.014 and 0.045 respectively). The presence of hypertension, diabetes, and renal insufficiency (serum creatinine above 1.2 mg/dL) showed a trend towards statistical significance (*p* = 0.051, 0.106, and 0.16 respectively).

**Table 1:** Characteristics of 15 patients with hypercalcemia attributable to calcium and vitamin D supplements.

No.	Age	Sex	Race	Medical Problems	Presenting Features	Meds	Supplements	Adm. Ca mg/dL	Last Ca mg/dL	PTH mg/mL	Ser. Cr mg/dL	Last Cr mg/dL	Bic mg/dL	Last Bic mg/dL	Ph mg/dL	Treatment
1	67	M	B	hypertension	abdominal pain	Simvastatin aspirin	Calcium Vit D3	12.4	10.1	2.8	1.6	0.7	37	29	3.8	IV fluids
2	75	F	W	Osteoporosis, hypertension	polyuria, weakness, bone pain	Lisinopril Metoprolol	Calcium 2 mg daily ibandronate	16.2	8.8	12.5	2.2	0.9	37	23	2.8	IV fluids, calcitonin
3	74	M	W	diabetes, hypertension, CAD renal insufficiency	mental status change, CVA	Metoprolol Lisinopril glipizide	calcium supplement multivitamin with mineral Vit D 1000 IU	10.9	10.3	12.4	2.4	2.4	33	29	4.6	IV fluids
4	60	M	B	hypertension	abdominal pain, abdominal swelling	Hydralazine testosterone	Calcium vitamin D 2000 IU per day	10.7	10.3	21.7	3.9	1.9	37	17	3.3	IV fluids
5	86	M	W	hypertension	abdominal pain, dehydration, weakness, back pain	Amlodipine Labetalol aspirin	Calcium multivitaminvit D	11.3	10.1	4.1	2.1	1.6	32	27	2.1	IV fluids
6	73	F	W	diabetes, hyperthyroidism	nausea and/or vomiting, cough, SOB	Methimazole metformin	calcium supplement Vit D multivitamin	11.6	9.0	<2.5	0.9	0.8	33	33	2.7	IV fluids
7	72	F	W	diabetes, hypertension, CAD	generalized edema, chronic pain	Simvastatin furosemide	Calcium supplement	13.8	10.4	<2.5	2.6	1.5	38	26	2.7	IV fluids, zoledronic acid
8	54	F	W	diabetes, hypertension, CAD renal insufficiency	abdominal pain, urinary retention, constipation	Nitroglycerin Amiodarone furosemide	TUMS antacid 4–6 mg per day	14.1	8.8	2.8	2.1	2.1	37	25	4.1	IV fluids, calcitonin
9	77	F	W	diabetes, hypertension	weakness	Insulin Losartan aspirin	Calcium multivitamin	14.5	9.0	3.6	2.2	1.5	33	25	2.6	IV fluids, calcitonin
10	65	M	W	diabetes, hypertension, CAD	nausea and/or vomiting, chest pain	Metformin glipizide Metoprolol mag ox	Multivitamin “large amount” of TUMS	15.0	7.6	8.0	1.6	1.0	35	28	4.2	IV fluids
11	55	F	W	diabetes, hypertension, renal insufficiency	nausea and/or vomiting, chills, dizziness	Insulin Enalapril Furosemide aspirin	Multivitamin Vit D antacid	14.6	8.7	53.1	3.9	2.6	32	20	2.6	IV fluids, calcitonin
12	83	F	W	hypertension	dyspnea, hypoxia	Atorvastatin Metoprolol Furosemide Hctz aspirin	calcium supplementmultivitamin Vit D	13.7	7.7	<2.5	1.4	1.0	43	30	2.7	IV fluids
13	60	F	B	diabetes, hypertension	pain from wounds	Insulin Valproic acid	Calcium Vit D 50000 IU weekly	13.8	9.1	7.8	2.7	1.1	33	24	4.1	IV fluids, zoledronic acid
14	68	M	B	diabetes, hypertension renal insufficiency	hypercalcemia	Hydralazine Levothyroxine Lisinopril amlodipine	calcium supplement TUMS antacid Vit D	12.7	9.3	<2.5	3.6	3.2	33	23	3.5	IV fluids
15	87	F	W	Hypertension Arthritis GERD Venous thromosis	encephalopathy, weakness, fatigue	Furosemide Amlodipine Warfarin albuterol	calcium supplemen Vit D 1000 IU multivitamin	13.8	9.7	<2.5	2.6	1.0	32	25	4.0	IV fluids

Meds: medications on presentation; Ca: serum calcium; PTH: parathyroid hormone level; Cr: serum creatinine; bic: serum bicarbonate; Ph: serum phosphorus.

**Table 2:** Prevalence of patient variables in subjects with and without CSS.

Variable	CSS (*N* = 15) *n* (%)	Non-CSS (*N* = 57) *n* (%)	*p*-Value
Gender (Female)	9 (60.0)	24 (42.1)	0.216
Calcium Intake	12 (80.0)	8 (14.0)	<0.0001
Vitamin D Intake	9 (60.0)	15 (26.3)	0.014
Multivitamin Use	7 (46.7)	12 (21.1)	0.045
Hypertension	14 (93.3)	39 (68.4)	0.051
Diabetes	9 (60.0)	21 (36.84)	0.106
Renal insufficiency (serum creatinine above 1.3 mg/dL)	14 (93.3)	44 (77.2)	0.160

## 5. Discussion

The ingestion of large amounts of calcium and absorbable alkali is a prime precipitating factor for the development of MAS. The disorder was originally described in association with the use of milk and sodium bicarbonate for the treatment of peptic ulcer disease [5]. The modern clinical presentation of this complex disorder could aptly be called the “calcium supplement syndrome” (CSS). The two faces of this syndrome are contrasted in Table 3. Although traditional MAS was described predominantly in middle-aged men, CSS appears to be more common in women [6]. Patients often have non-specific symptoms, and hypercalcemia and renal insufficiency may be incidentally noted. Recovery is the rule with rehydration and discontinuation of calcium and vitamin D [10]. Concurrent bisphosphonate therapy may predispose to post-treatment hypocalcemia because of suppressed bone turnover [7].

**Table 3:** A comparison of the traditional and modern-day presentations of the milk-alkali syndrome (or “calcium supplement syndrome”).

	Milk-Alkali Syndrome	Calcium Supplement Syndrome
Patient population	Middle-aged men	Older women
Symptoms	Present	Asymptomatic
Presentation	Acute: nausea, vomiting, weakness, mental status changes	Hypercalcemia and renal insufficiency are incidental findings
	Chronic: polyuria, polydipsia, muscle aches	
Phosphorus level	Normal or high due to phosphate load from milk	Low from lack of milk load and the binding properties of calcium carbonate
Post-treatment hypocalcemia	Not usually seen	May develop
Prognosis	Variable	Complete recovery with treatment (insufficient data)

We undertook a retrospective, records-based study to ascertain the prevalence and characteristics of CSS in hypercalcemic patients hospitalized over a three-year period. Our investigation revealed that the clinico-biochemcial diagnosis of CSS was present in one-fifth of hospitalized patients with PTH-independent hypercalcemia. Older age seemed to be a risk factor (mean age 70.4 years). Hypertension, diabetes, and renal insufficiency were more prevalent in CSS patients compared with hypercalcemic patients without CSS. As expected, all patients with CSS were taking calcium supplements, and a majority was taking vitamin D and multivitamins—these three factors being statistically significantly associated with CSS in our analysis. It is worth noting that only one patient was on a medication known to aggravate hypercalcemia (hydrochlorthiazide) [11]. Intravenous hydration was administered in all patients, supplements were discontinued, and 40% of the patients received calcium-lowering drug therapy.

The presence of all four criteria (renal insufficiency, metabolic alkalosis, elevated serum bicarbonate, and history of calcium/vitamin D ingestion) for diagnosis of calcium supplement syndrome was purposely done to make the clinical diagnosis as stringent and accurate as possible. This minimized and hopefully eliminated chance or coincidental occurrence, which would be unlikely. It would also substantially reduce the possibility of comorbidities as the sole cause of the presentation, as would the reversal of the abnormalities with appropriate therapy.

Our results reinforce a number of risk factors that may predispose to CSS, namely calcium ingestion, older age, volume depletion, and renal insufficiency with reduced glomerular filtration rate (GFR). The risks associated with calcium preparations are often assumed by patients as being minimal, with many patients likely taking high daily doses under the maxim that “more is better” [12,13]. Chronic health conditions and the use of medications such as thiazide diuretics, angiotensin converting enzyme inhibitors, angiotensin receptor blockers, and non-steroidal anti-inflammatory drugs (NSAIDs) have also been implicated [6].

It has been suggested that inadequate suppression of calcitriol in some individuals leads to intestinal hyperabsorption in response to large amounts of calcium ingestion, resulting in hypercalcemia [14,15]. The latter may predispose to renal vasoconstriction and acute kidney injury [16]. In this context, the presence of limited renal reserve appears to be a strong risk factor in the pathogenesis of CSS. The ensuing urinary sodium loss results in volume depletion, stimulating tubular absorption of bicarbonate. Thus, the combined effects of increased alkali intake, volume depletion, and decreased GFR results in metabolic alkalosis [17]. Volume contraction due to vomiting or diuretics worsens both hypercalcemia and alkalosis. In addition, older patients may have a reduced skeletal buffer against hypercalcemia [6].

## 6. Conclusions

CSS is of potentially significant public health importance. Calcium and vitamin D supplementation is thought to have general health and preventive benefits, and is also recommended for the treatment of osteoporosis and vitamin D deficiency. However, it is becoming evident that liberal calcium intake in susceptible individuals may not be entirely benign, with consequences not unlike the classic milk-alkali syndrome of the past. Awareness of its health implications should alert patients and clinicians alike to the dangers of excessive or indiscriminate use of calcium and vitamin D supplements.
